# Resolution of dysphagia following endoscopic resection of a large, nodular, esophageal inlet patch with low grade dysplasia

**DOI:** 10.1055/a-2302-7690

**Published:** 2024-04-29

**Authors:** Katarzyna M. Pawlak, Christopher Teshima

**Affiliations:** 1 Division of Gastroenterology, St. Michael’s Hospital, University of Toronto, Toronto, Canada

A 44-year-old man was referred due to chronic dysphagia to solids and a persistent globus sensation. Esophagogastroduodenoscopy (EGD) revealed a nodular inlet patch with a hyperplastic polypoid appearance occupying 70% of the luminal circumference in the proximal esophagus from 18 to 20 cm. Narrow-band imaging did not reveal dysplastic features and biopsies confirmed gastric heterotopia without dysplasia. Further work-up with barium swallow, chest X-ray, esophageal manometry, and pH testing were normal. Medical therapy with twice-daily proton pump inhibitor did not improve his symptoms. As such, endoscopic resection was proposed.

The procedure was performed under general anesthesia with endotracheal intubation ( Video 1 ). Piecemeal endoscopic mucosal resection (EMR) was performed using a Duette multiband mucosectomy device (Cook Medical, Limerick, Ireland) after submucosal lifting using normal saline with diluted methylene blue. The entire inlet patch was successfully removed using nine bands with minor bleeding controlled using hemostatic forceps. Diluted triamcinolone (34 mg) was injected into the EMR defect for stricture prophylaxis, followed by treatment with budesonide slurry and sucralfate suspension for 4 weeks. The pathology revealed gastric oxyntic mucosa with low grade dysplasia. Follow-up EGD at 1 year confirmed the absence of any residual inlet patch and no dysplastic findings. Crucially, the patient had complete resolution of his chronic symptoms.

Successful endoscopic resection with multiband mucosectomy device of a large inlet patch with low grade dysphagia causing dysphagia.Video 1

The inlet patch is a congenital anomaly with a prevalence of up to 1% 1 . Most inlet patches are asymptomatic but occasionally may cause dysphagia or globus sensation 2 . They are usually ignored by most endoscopists due to small size, but the finding of low grade dysplasia in our case highlights the importance of close examination and consideration for endoscopic intervention when there are abnormal features. Small, symptomatic inlet patches can be treated by EMR or mucosal ablation 3 4 , whereas larger or polypoid inlet patches may be completely and safely removed by multiband mucosectomy, as demonstrated by our case.

Endoscopy_UCTN_Code_CCL_1AB_2AC_3AD

## Notes

Endoscopy E-Videos https://eref.thieme.de/e-videos
*E-Videos* is an open access online section of the journal *Endoscopy* , reporting on interesting cases and new techniques in gastroenterological endoscopy. All papers include a high-quality video and are published with a Creative Commons CC-BY license. Endoscopy E-Videos qualify for HINARI discounts and waivers and eligibility is automatically checked during the submission process. We grant 100% waivers to articles whose corresponding authors are based in Group A countries and 50% waivers to those who are based in Group B countries as classified by Research4Life (see: https://www.research4life.org/access/eligibility/ ). This section has its own submission website at https://mc.manuscriptcentral.com/e-videos .
